# New Hampshire Veterinary Medical Association

**Published:** 1896-03

**Authors:** L. Pope

**Affiliations:** Secretary


Secretary.

### NEW HAMPSHIRE VETERINARY MEDICAL ASSOCIATION.

THE ninth meeting was held in the Eagle Hotel, Concord, on February 4th. The meeting was called to order at 11.30 A.M., by Dr. Russell. Minutes of the previous meeting were then read and accepted. The following answered to the roll-call: Drs. Russell, Macguire, Abbott, Wilkinson, Lilico, and Pope.

Dr. Russell then followed by a short address concerning the usefulness of the Association, the necessity of attending its meetings, etc., and moved that the by-laws be changed relative to the time of meetings, making them quarterly instead of monthly. Seconded by Dr. Wilkinson, and carried unanimously.. The Secretary was instructed by vote of the Association to read the communication from the Army Legislation Committee of the United States Veterinary Medical Association, including letter, bill proposed, and reasons why the veterinary service of the army should be improved. This subject being fully discussed, the Secretary was asked to send resolutions and copy of the bill to each and every member of Congress coming from New Hampshire. No further business to come before the meeting, Dr. Russell was called and read a very interesting paper on "Heredity," treating the subject from a human as well as an equine standpoint, their similarity rendering the subject very instructive. Discussion followed, showing many differences, and bringing out many interesting points.

Meeting adjourned at 1.30 P.M. until May. L. POPE, JR., M.D.V.,
Secretary.

